# Soluble CD83 modulates human-monocyte-derived macrophages toward alternative phenotype, function, and metabolism

**DOI:** 10.3389/fimmu.2023.1293828

**Published:** 2023-12-14

**Authors:** Katrin Peckert-Maier, Andreas B. Wild, Laura Sprißler, Maximilian Fuchs, Philipp Beck, Jean-Philippe Auger, Pia Sinner, Astrid Strack, Petra Mühl-Zürbes, Ntilek Ramadan, Meik Kunz, Gerhard Krönke, Lena Stich, Alexander Steinkasserer, Dmytro Royzman

**Affiliations:** ^1^ Department of Immune Modulation, Universitätsklinikum Erlangen, Friedrich– Alexander Universität Erlangen–Nürnberg, Erlangen, Germany; ^2^ Fraunhofer Institute for Toxicology and Experimental Medicine (ITEM), Hannover, Germany; ^3^ Department of Internal Medicine 3 – Rheumatology and Immunology, Friedrich-Alexander University Erlangen-Nürnberg (FAU) and Universitätsklinikum Erlangen, Erlangen, Germany; ^4^ Chair of Medical Informatics, Friedrich-Alexander-Universität Erlangen-Nürnberg (FAU), Erlangen, Bavaria, Germany

**Keywords:** soluble CD83, human-monocyte-derived macrophages, alternative activation, checkpoint molecule, LXR pathway

## Abstract

Alterations in macrophage (Mφ) polarization, function, and metabolic signature can foster development of chronic diseases, such as autoimmunity or fibrotic tissue remodeling. Thus, identification of novel therapeutic agents that modulate human Mφ biology is crucial for treatment of such conditions. Herein, we demonstrate that the soluble CD83 (sCD83) protein induces pro-resolving features in human monocyte-derived Mφ biology. We show that sCD83 strikingly increases the expression of inhibitory molecules including ILT-2 (immunoglobulin-like transcript 2), ILT-4, ILT-5, and CD163, whereas activation markers, such as MHC-II and MSR-1, were significantly downregulated. This goes along with a decreased capacity to stimulate alloreactive T cells in mixed lymphocyte reaction (MLR) assays. Bulk RNA sequencing and pathway analyses revealed that sCD83 downregulates pathways associated with pro-inflammatory, classically activated Mφ (CAM) differentiation including HIF-1A, IL-6, and cytokine storm, whereas pathways related to alternative Mφ activation and liver X receptor were significantly induced. By using the LXR pathway antagonist GSK2033, we show that transcription of specific genes (e.g., *PPARG*, *ABCA1*, *ABCG1*, *CD36*) induced by sCD83 is dependent on LXR activation. In summary, we herein reveal for the first time mechanistic insights into the modulation of human Mφ biology by sCD83, which is a further crucial preclinical study for the establishment of sCD83 as a new therapeutical agent to treat inflammatory conditions.

## 1 Introduction

Macrophages (Mφ) play a vital part in defense against invading pathogens and in maintaining tissue homeostasis since they contribute to induction of inflammatory responses but also to tissue regeneration upon injury. This variety of different tasks within the human body requires a high level of heterogeneity and functional plasticity. Consequently, Mφ are very dynamic cells that integrate signals from the given microenvironment and react by adopting different phenotypes and functions. Although the diverse phenotypes of Mφ should be seen very plastic and adaptable, Mφ are often classified into two polar extremes of activation: pro-inflammatory, classically activated Mφ (CAM) and anti-inflammatory, alternatively activated Mφ (AAM). However, the strict dichotomy of Mφ into two groups of inflammatory CAM and resolving AAM does not live up to the enormous heterogeneity of this cell type and this stigma has been challenged since it was proposed. The heterogeneity of human Mφ biology becomes even clearer by a recently published study, which identified 10 different clusters of activation states after treatment of human monocyte-derived Mφ with 28 distinct stimulations (1).

Thus, Mφ polarization leads to specific transcriptional profiles, expression of specific surface markers, and secretion of cytokines. One crucial process, which modulates and orchestrates these functionally distinct activation states of human Mφ, is the regulation of cell metabolism (2). Upon tissue injury, pathogen-associated molecular patterns (PAMPs) or damage-associated molecular patterns cause polarization into pro-inflammatory CAM during the initial phase of an inflammatory response. This activation is linked to increased expression of surface molecules (MHC-II/CD86/MSR-1), enhanced production of reactive oxygen species (ROS), pro-inflammatory cytokines (TNF-α, IL-6, MCP-1), and rewiring of cellular metabolism toward glycolysis (3, 4). Glycolytic genes including glucose transporters, such as GLUT1, are promptly downregulated at days 2–3 after tissue injury, correlating with a conversion into resolving AAM with a specific phenotype and metabolic profile. AAM polarization can be induced by glucocorticoids, IL-4, IL-13, or IL-10. The AAM state is characterized by activation of specific surface receptors (e.g., CD163, immunoglobulin-like transcript (ILT)-2, ILT-4, ILT-5, and CD36) (4, 5), effector molecules/cytokines (e.g., IL-4, TGF-β, IL-10, Arginase-1) and transcription factors, including Krüppel-like factor 4 (KLF-4), GATA binding protein-3 (GATA3), peroxisome proliferator-activated receptor gamma (PPARγ), and liver X receptors (LXR) (2, 5, 6). This change in the transcriptomic and metabolomics profile is essential for resolution of inflammation and subsequent tissue repair. The metabolic state of anti-inflammatory AAM is dictated by transcription factors like KLF-4, PPARγ, and LXR and is characterized by increased oxidative phosphorylation (OXPHOS), fatty acid oxidation (FAO), and glutaminolysis (7). Thus, in contrast to the highly glycolytic state of CAM, AAM metabolism is fueled by lipid anabolism and OXPHOS.

In previous studies, we have shown that the CD83 molecule is a gatekeeper for cellular activation in murine microglia and Mφ, promoting the stabilization of an AAM phenotype. Deletion of CD83 in the respective cell types leads to transition toward an inflammatory cell type, identifying CD83 as an important checkpoint molecule that contributes to resolution of inflammation in the murine system (8, 9). The CD83 protein exists in two isoforms: a membrane-bound (mCD83) and a soluble form (sCD83), largely consisting of the extracellular domain of the mCD83 protein (10, 11). We have already demonstrated a resolving mode of action by sCD83 in murine corneal transplantation as well as skin wound healing by induction of pro-resolving Mφ (12, 13). In addition, in a very recent study, Gong et al. confirmed the immunomodulatory effect of sCD83 in pig alveolar Mφ by inducing AAM rather than CAM polarization (14). In 2017, Horvatinovich et al. identified myeloid differentiation factor (MD-2), the coreceptor within the TLR-4/MD-2 receptor complex, as the binding partner for sCD83 on human CD14+ monocytes (15). However, translational investigations on the impact of sCD83 on human Mφ biology have not been conducted so far. In this study, we reveal for the first time that human monocyte-derived Mφ differentiation *in vitro* in the presence of sCD83 leads to striking phenotypical, functional, and metabolic changes. We demonstrate that sCD83-differentiated human Mφ showed increased expression of inhibitory markers, such as ILT-2, ILT-4, ILT-5, and CD163, whereas expression of activation marker MSR-1 as well as MHC-II was significantly decreased, resulting in impaired T-cell stimulatory capacity. Bulk RNA sequencing (RNA-seq) analyses revealed significant changes in the transcriptome of sCD83-differentiated human monocyte-derived Mφ, which are linked to alternative activation of Mφ and LXR signaling. We verified these data on the mRNA as well as on the protein level and reveal significant induction of factors associated with AAM, such as KLF-4 or PPAR-γ. Functional studies revealed significant reduction in lipid uptake of sCD83-treated human monocyte-derived Mφ. In summary, we report for the first time data regarding sCD83 in human Mφ biology, which is a further preclinical step using sCD83 as a therapeutic agent for the treatment of inflammatory conditions.

## 2 Results

### 2.1 Soluble CD83 strikingly modulates the expression of surface receptors on human Mφ

**Figure 1:**
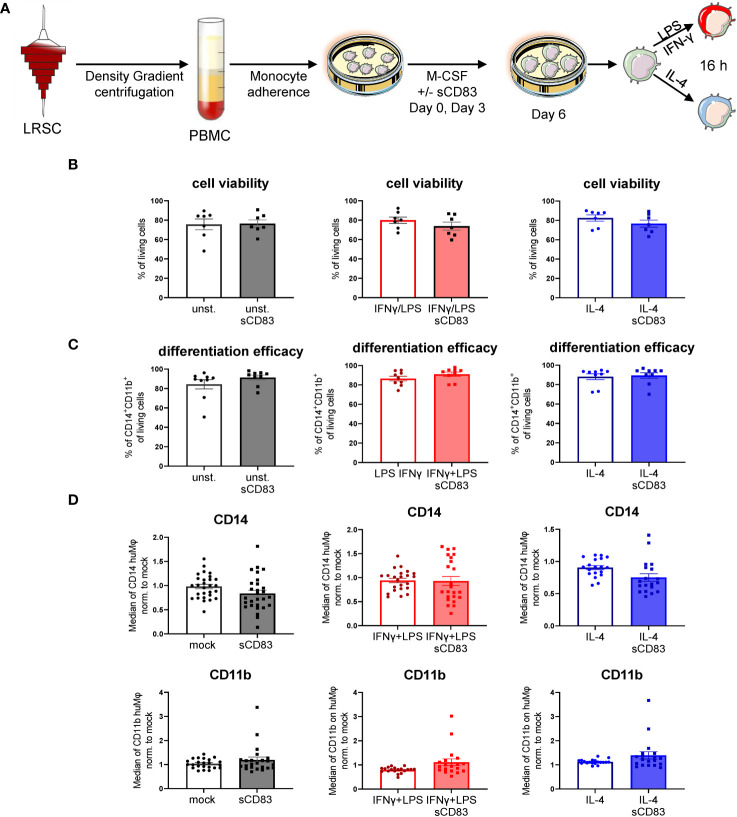
Soluble CD83 does not interfere with viability nor differentiation efficacy of human Mφ. **(A)** Experimental set up to check whether sCD83 affects cell viability or differentiation efficacy of human monocyte-derived Mφ. PBMCs were isolated via density gradient centrifugation and subsequently monocytes were seeded for adherence. Mφ were differentiated from monocytes in the presence of M-CSF (20 ng/ml) and sCD83 (25 µg/ml) or the corresponding amount of DPBS was added on day 0 as well as day 3 during the differentiation process. Mφ were subsequently seeded and polarized via LPS (100 ng/ml) +IFN-γ (300 U/ml), or IL-4 (20 ng/ml). **(B)** Cell viability assessment of human monocyte-derived Mφ generated +/-sCD83 by flow cytometry. **(C)** sCD83 has no influence on differentiation efficacy of human monocyte-derived Mφ **(D)** Assessment of expression levels of CD14 (upper row) as well as CD11b (lower row) are not influenced by sCD83 when present during Mφ differentiation. Statistical analyses were performed by One-way ANOVA or the appropriate corresponding non-parametric test. Data are represented as mean ± SEM. Experiments were performed at least three times. One dot per bar graph represent one donor. The absence of asterisks indicates that there is no statistical significance.

Previous studies using murine bone-marrow-derived Mφ differentiated in the presence of sCD83 reported the capacity to induce an alternative activation state in Mφ, which improved skin wound healing and induced tolerance in corneal transplantation *in vivo* (12, 13). However, the effect of sCD83 on human Mφ biology has not yet been described. Therefore, we generated human monocyte-derived Mφ from blood of healthy donors in the presence of sCD83, or the corresponding amount of DPBS as a control. On day 6 of differentiation, Mφ were harvested and subsequently stimulated using pro-inflammatory mediators (LPS+IFNγ) or alternatively activating stimuli (IL-4) for 16 h (**Figure 1A**). At first, we analyzed whether sCD83 administration has any effect on cell viability or differentiation efficacy. The gating strategy for the flow cytometric analyses of human monocyte-derived Mφ is depicted in **Supplementary Figure 1** (S1). As depicted, administration of sCD83 to human Mφ differentiation does not interfere with viability of the cells (**Figure 1B**) nor differentiation efficacy (**Figure 1C**) regardless of the subsequent stimulation. In addition, expression levels of CD14 (**Figure 1D**, upper row) and CD11b (**Figure 1D**, lower row) on CD11b^+^CD14^+^ human Mφ were not affected when sCD83 was added to the human Mφ differentiation process. From these data, we concluded that administration of sCD83 to human monocyte-derived Mφ has no effect on the differentiation process of Mφ or on cell viability.

**Figure 2:**
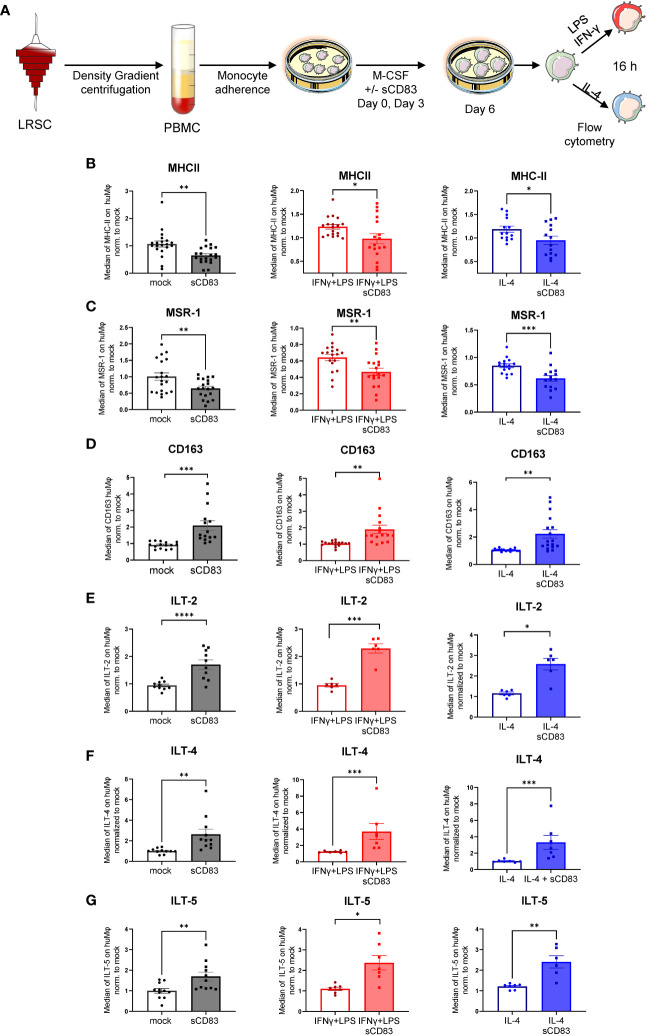
Soluble CD83 strikingly modulates the phenotype of human monocyte-derived Mφ. **(A)** Experimental setup for the analyses of the phenotype of Mφ differentiated in the presence of sCD83 or PBS (referred to as mock). PBMCs were isolated via density gradient centrifugation, and subsequently, monocytes were seeded for adherence. Monocytes were differentiated into Mφ in the presence of M-CSF (20 ng/ml), +/− sCD83 (25 µg/ml). sCD83 was added on day 0 as well as day 3 during the differentiation process. Mφ were subsequently seeded and polarized via LPS (100 ng/ml) +IFN-γ (300 U/ml), or IL-4 (20 ng/ml) for 16 h. Subsequently, human monocyte-derived Mφ were analyzed by flow cytometry for the expression of **(B)** MHC-II, **(C)** MSR-1, **(D)** CD163, **(E)** ILT-2, **(F)** ILT-4, and **(G)** ILT-5. Data are represented as mean ± SEM. Statistical analysis was performed using a Mann–Whitney U test. Experiments were performed at least three times. One dot per bar graph represents one donor. n.s., not significant, which indicates there is no statistical significance; *p< 0.05; **< 0.01; *** p< 0.001; **** p< 0.0001.

Next, we analyzed the surface receptor expression profile by flow cytometry. An overview of the experimental setup is depicted in **Figure 2A**. Interestingly, sCD83-differentiated Mφ exhibited significantly less MHC-II surface expression compared with mock-differentiated control Mφ regardless of the preceding stimulation (**Figure 2B**). By contrast, the costimulatory molecule CD86 was unaffected by sCD83 treatment (data not shown). In addition, we detected significantly decreased levels of activation marker MSR-1 on the surface of human Mφ regardless of the preceding stimulation (**Figure 2C**). Notably, the scavenger receptor CD163, which is known to be expressed on regulatory human Mφ upon glucocorticoid as well as IL-10 stimulation *in vitro* (16), was significantly upregulated when sCD83 was present during differentiation (**Figure 2D**). Impressively, anti-inflammatory molecules of the immunoglobulin-like transcript family, including ILT-2 (**Figure 2E**), ILT-4 (**Figure 2F**), and ILT-5 (**Figure 2G**), were significantly upregulated on sCD83-treated Mφ. Interestingly, none of the observed changes depended on the subsequent stimulation, suggesting a durably imprinted effect of sCD83 on Mφ differentiation leading to a stable regulatory phenotype.

### 2.2 Soluble CD83 impairs the T-cell stimulatory capacity of human-monocyte-derived Mφ

**Figure 3:**
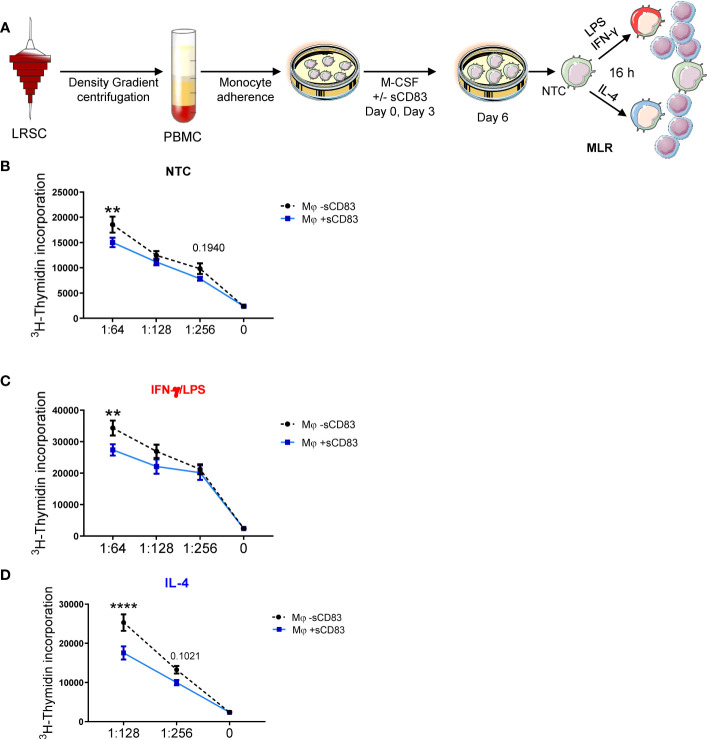
Differentiation of human monocyte-derived Mφ in the presence of sCD83 results in less T cell stimulatory capacity. **(A)** Experimental set up for the analyses of the allogeneic T cell stimulatory capacity of human Mφ differentiated in the presence of sCD83 or PBS (referred to as mock). PBMCs were isolated via density gradient centrifugation and subsequently monocytes were seeded for adherence. Monocytes were differentiated into Mφ in the presence of M-CSF (20 ng/ml), +/- sCD83 (25 µg/ml). sCD83 was added on day 0 as well as day 3 during the differentiation process. Mφ were subsequently seeded and polarized LPS (100 ng/ml) +IFN-γ (300 U/ml), or IL-4 (20 ng/ml). Subsequently, human monocyte-derived Mφ were analyzed for the capacity to stimulate alloreactive T cells using MLR assays. **(A–D)** Human monocyte-derived Mφ Untreated **(B)**, LPS+IFNγ **(C)** IL-4 **(D)** stimulated sCD83-differentiated human Mφ show decreased capacity to stimulate alloreactive T cells regardless of the preceding stimulation compared to control Mφ N ≥ 7; Experiments were performed at least 4 times for each stimulation. Data are represented as mean ± SEM. Statistical analysis was performed using a Two-way ANOVA or the appropriate corresponding non-parametric test. Experiments were performed at least three times. One dot per bar graph represent one donor n.s., not significant, which indicates there is no statistical significance; ** < 0.01; **** p < 0.0001.

Since we have observed a rather regulatory phenotype of sCD83-differentiated Mφ, we next performed functional assay to analyze whether this translates into disturbed stimulatory capacity toward T cells. Thus, we generated human monocyte-derived Mφ in the presence of sCD83 and subsequently stimulated them with LPS+IFN-γ or IL-4, or left Mφ untreated for 16 h. The next day, we added responder T cells from allogeneic donors to Mφ for MLR assays. An overview of the experimental setup is depicted in **Figure 3A**. As shown in **Figures 3B–D**, we observed a significant reduction in allogeneic T-cell proliferation when Mφ were differentiated in the presence of sCD83 regardless of the following stimulation. From these data, we conclude that sCD83-induced phenotypic changes have also functional relevance by subverting the ability of human monocyte-derived Mφ to promote T-cell proliferation.

### 2.3 Soluble CD83 drastically changes the transcriptome in human monocyte-derived Mφ by inducing transcripts linked to alternative and liver X receptor activation

**Figure 4:**
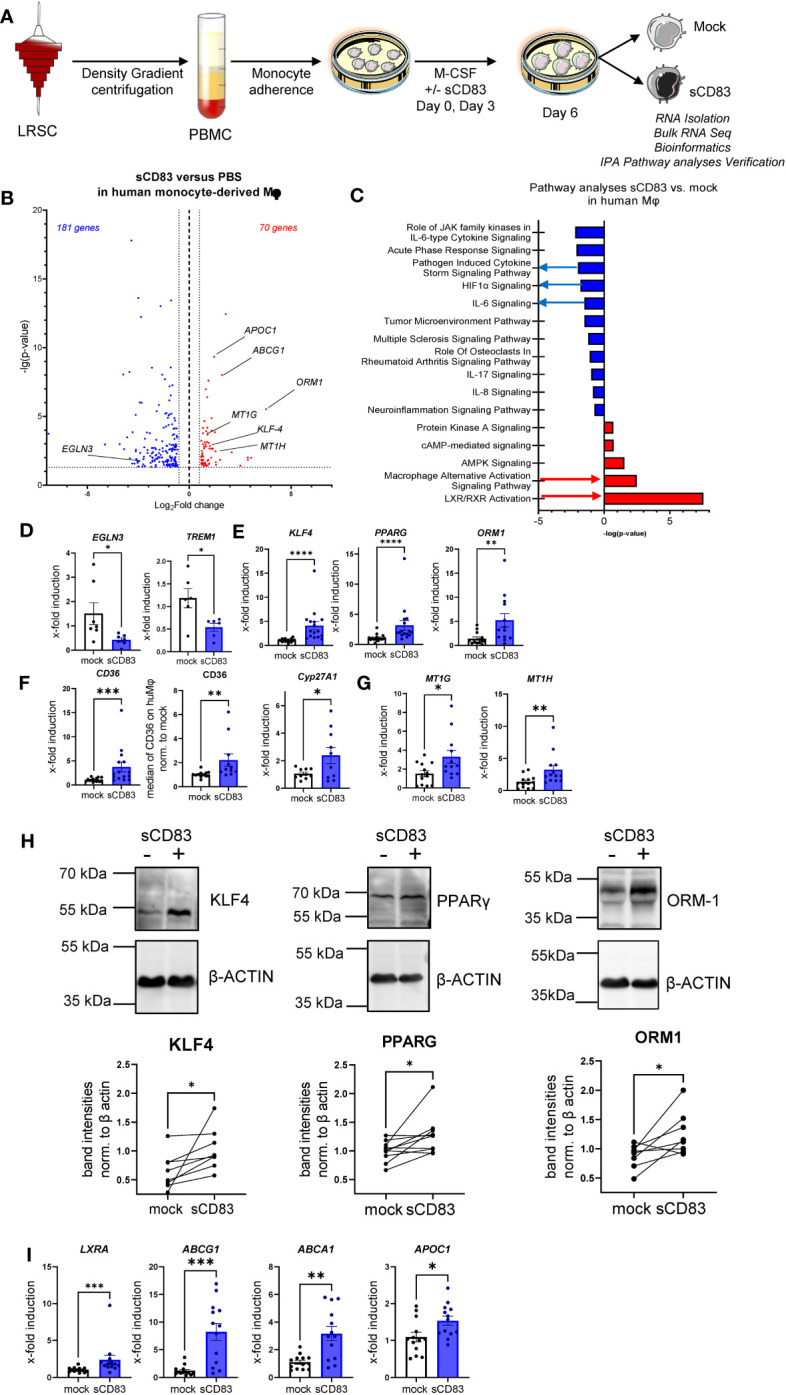
Soluble CD83-differentiated human monocyte-derived Mφ show distinct profile changes toward alternative activation on mRNA as well as on the protein level. **(A)** Experimental setup for the bulk RNA sequencing experiment and verification on mRNA as well as on the protein level by qPCR and Western blot. PBMCs were isolated via density gradient centrifugation, and subsequently, monocytes were seeded for adherence. Monocytes were differentiated into Mφ in the presence of M-CSF (20 ng/ml) and +/− sCD83 (25 µg/ml). sCD83 was added on day 0 as well as day 3 during the differentiation process. Subsequently, Mφ were harvested on day 6 of differentiation and RNA sequencing analyses as well as verification on protein as well as mRNA levels were performed by Western blotting as well as qPCR. **(B)** Volcano plot of RNA sequencing analyses of human monocyte-derived Mφ differentiated in the presence of sCD83 versus mock-treated Mφ (n = 3). On the right-hand side of the logFold change 0 value, significantly upregulated gene transcripts (red) are displayed, whereas the dots on the left-hand side represent significantly downregulated transcripts (blue) with logFC ≥0.585, respectively. On the Y-axis, the p-value is displayed. **(C)** Transcriptomic data were analyzed using the Ingenuity Pathway Analysis (IPA) software from QIAGEN’s revealing downregulation of inflammatory pathways and enhanced upregulation of resolving Mφ pathways including alternative activation as well as LXR activation. **(D)** qPCR analyses for mRNA expression analyses of HIF-1A target genes *EGLN3* and *TREM-1*, which are associated with CAM polarization in +/−sCD83 differentiated Mφ. **(E)** qPCR analyses for mRNA expression analyses of genes, such as *KLF-4*, *PPARγ*, and *ORM1*, which are associated with AAM in +/−sCD83-differentiated Mφ. **(F)** qPCR/flow cytometric analyses for PPARγ target genes, such as *CD36*, CD36, and *Cyp27A1*, which are associated with AAM in +/−sCD83-differentiated Mφ. **(G)** qPCR analyses for mRNA expression analyses for *MT1G* as well as *MT1H*, which are associated with anti-inflammatory properties. **(H)** Western blot analyses of whole-cell lysates of human Mφ-differentiated +/− sCD83 for assessment of KLF-4, PPARG, and ORM-1 protein levels. Quantification of Western blots was performed using the ImageJ software and β-ACTIN served as loading control. **(I)** qPCR analyses for mRNA expression analyses for LXRa (*NR1H3*) and its target genes, such as *ABCG1*, *ABCA1*, and *APOC1*, which are associated with AAM in +/− sCD83-differentiated Mφ. Data are represented as mean ± SEM. Statistical analysis was performed using a one-way ANOVA or the appropriate corresponding non-parametric test. Experiments were performed at least three times. One dot per bar graph represents one donor. n.s., not significant, which indicates there is no statistical significance; *p< 0.05; **< 0.01; ***p< 0.001; ****p< 0.0001.

We have demonstrated that sCD83 drives Mφ differentiation into an immunoregulatory phenotype. Next, we aimed to scrutinize the underlying mechanisms and thus performed bulk RNA sequencing analyses to unravel sCD83-induced molecular mechanisms and signaling pathways. As depicted in **Figure 4A**, we isolated RNA from human monocyte-derived Mφ that were differentiated in the presence of sCD83 or PBS as a control. Our transcriptomic data revealed 251 differentially regulated genes in sCD83-treated Mφ compared with PBS-treated Mφ (**Figure 4B**). We found 181 significantly downregulated gene transcripts and 70 upregulated gene transcripts in sCD83-differentiated Mφ. Among upregulated transcripts, we identified genes, which are associated with alternative Mφ activation, such as *KLF-4*, *ORM-1*, and *ABCG1* (17, 18). To get a broader overview of affected cellular pathways, we further analyzed transcriptome data using the Ingenuity Pathway Analysis (IPA) software (**Figure 4C**). Pathway analyses of transcriptomic data from sCD83-differentiated Mφ revealed significant downregulation of inflammatory pathways associated with classical Mφ activation, e.g., pathogen-induced cytokine storm signaling pathway, IL-6 signaling, and HIF-1α signaling (**Figure 4C**, blue). As shown in **Figure 4E**, we observed a significant downregulation of HIF1α target gene *EGLN3* (**Figure 4D**, left bar graph) and a significant reduction of triggering receptor expressed on myeloid cells 1 (TREM-1) expression (**Figure 4D**, right bar graph) associated with inflammatory Mφ activation in sCD83-treated human monocyte-derived Mφ (19). Remarkably, pathways associated with resolving alternative Mφ activation and activation of the LXR/RXR pathway were significantly enhanced in sCD83-differentiated compared with control Mφ (**Figure 4C**). We further assessed expression of key components from both pathways using qPCR analyses (**Figures 4D, E**) and Western blot analyses (**Figure 4F**). We show that administration of sCD83 to Mφ differentiation results in a significant induction of transcription factors KLF4 as well as PPARG on mRNA (**Figure 4E**, first and second bar graphs) and on the protein level (**Figure 4H**, first and second graphs), which are essential for polarization of Mφ toward an alternative activation profile (20–22). In addition, we demonstrate upregulation of *ORM1* on both the mRNA level (**Figure 4E**, third bar graph) and on the protein level (**Figure 4H**, third bar graph), which is known to induce polarization of monocytes toward alternative CD163^+^ cells (18). Concomitantly, immunomodulatory metallothioneins, e.g., MT1G and MT1H, were also induced in Mφ, which were differentiated in the presence of sCD83 (**Figure 4G**), which is also in line with a previous study investigating the effect of sCD83 on osteoclast differentiation (23, 24). Next, we checked for the expression of PPARG target genes, e.g., *CYP27A1* and *CD36* (25), and detected significant upregulation of both transcripts *(* **Figure 4F**). We verified the increased CD36 expression also on the protein level using flow cytometry (**Figure 4F**). From these very interesting data, we concluded that sCD83 administration reprograms human Mφ toward an alternative activation profile. Since our bulk RNA sequencing analyses also revealed an activation of the LXR pathway in Mφ treated with sCD83 (**Figure 4C**), we next assessed the expression of transcription factor *NR1H3/LXRA* as well as selected and well-characterized target genes. We reveal that administration of sCD83 during Mφ differentiation results not only in significant induction of transcription factor *LXRA* but also in its direct induction of target genes ABCA1, ABCG1, and *APOC1* (26) (**Figure 4I**). From these data, we suggest that sCD83-induced effects are dependent on LXR signaling pathway activation. Furthermore, we conclude that administration of sCD83 to human monocyte-derived Mφ-differentiation leads to an alternative phenotypic and metabolic profile that may favor resolution of inflammatory responses.

### 2.4 Soluble CD83-mediated effects are partially dependent on LXR activation and sCD83-treated Mφ show less lipid load

**Figure 5:**
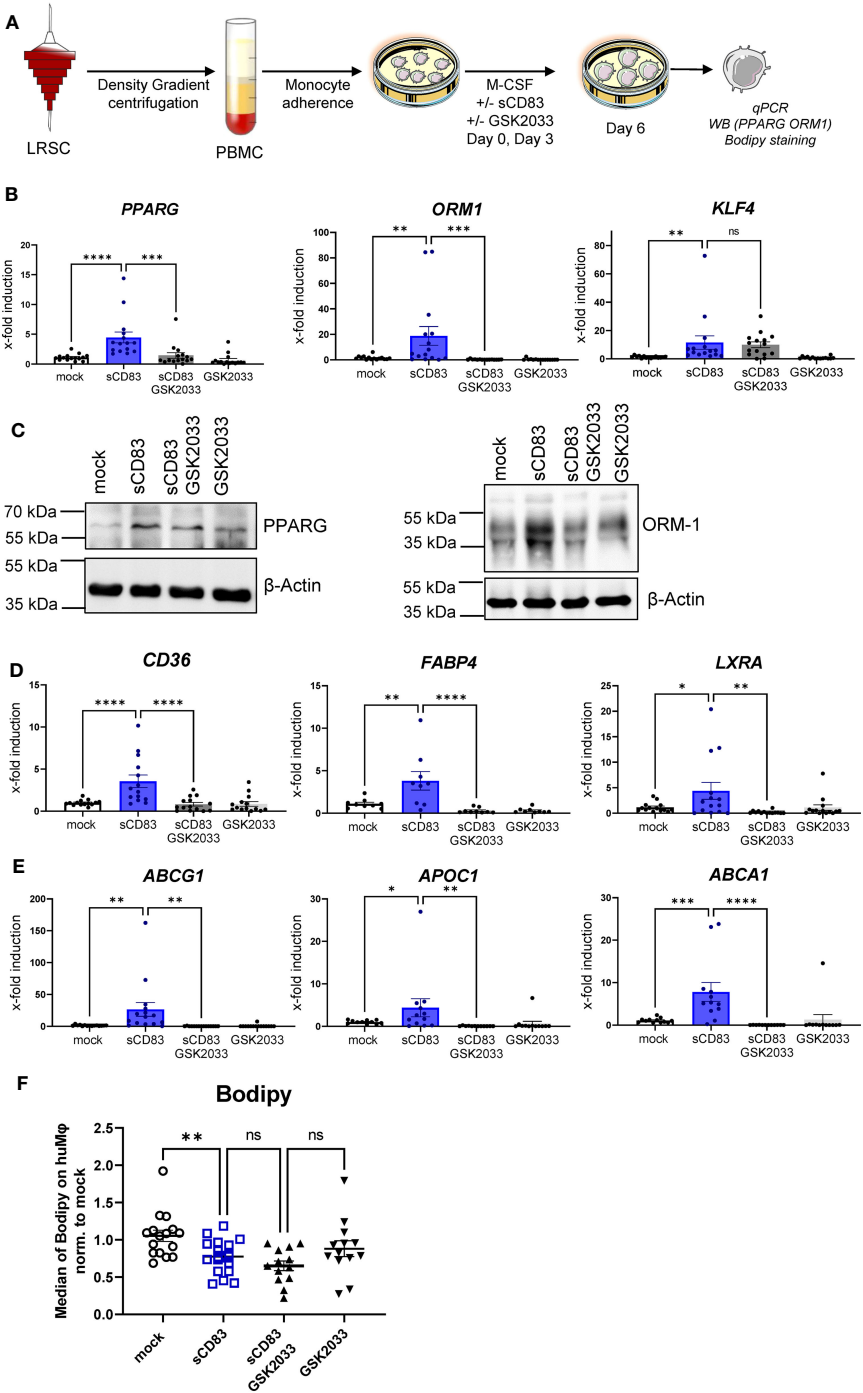
Soluble CD83-induced transcriptional changes are partly dependent on LXR pathway activation. **(A)** Experimental setup for the LXR pathway blocking experiments. PBMCs were isolated via density gradient centrifugation, and subsequently, monocytes were seeded for adherence. Monocytes were differentiated into Mφ in the presence of M-CSF (20 ng/ml) and +/− sCD83 (25 µg/ml). sCD83 was added on day 0 as well as day 3 during the differentiation process. GSK2033 (1 mM) was applied 1 h before sCD83 administration. Subsequently, cells were harvested for subsequent analyses. **(B)** qPCR analyses for mRNA expression of *PPARG* (first bar graph), *ORM-1* (second bar graph), and *KLF-4* (third bar graph) of mock-, sCD83-, sCD83 + GSK2033-, or GSK2033-treated Mφ. **(C)** Western blot analyses of whole-cell lysates of human monocyte-derived Mφ differentiated +/− sCD83 for assessment of PPARG as well as ORM-1 protein levels. **(D)** qPCR analyses for mRNA expression of *CD36* (first bar graph), *FABP4* (second bar graph), *LXRa* (third bar graph) of mock-, sCD83-, sCD83 + GSK2033-, or GSK2033-treated Mφ. **(E)** qPCR analyses for mRNA expression of *ABCG1* (first bar graph), *APOC1* (second bar graph), *ABCA1* (third bar graph) of mock-, sCD83-, sCD83 + GSK2033-, or GSK2033-treated Mφ. **(F)** Flow cytometric analyses using Bodipy staining, which is a fluorescent dye that stains lipid droplets in human monocyte-derived Mφ. Data are represented as mean ± SEM. Statistical analysis was performed using a one-way ANOVA or the appropriate corresponding non-parametric test. Experiments were performed at least three times. One dot per bar graph represents one donor. n.s., not significant, which indicates there is no statistical significance; *p< 0.05; **< 0.01; *** p< 0.001; **** p< 0.0001.

To advance the idea that sCD83-dependent effects on human monocyte- derived Mφ are dependent on activation of the LXR pathway, we performed additional experiments using the selective LXR antagonist GSK2033, which inhibits both LXRα- and LXRβ-mediated expression of target genes. The experimental setup for the LXR-pathway blocking experiment is depicted in **Figure 5A**. We generated human monocyte-derived Mφ from blood of healthy donors and sCD83, or the corresponding amount of PBS+DMSO as a control was added to the differentiation process on day 0 and day 3. The antagonist GSK2033 was applied 1 h before sCD83 administration. On day 6, we harvested human monocyte-derived Mφ for subsequent analyses. As in our previous experiments, we observed that sCD83 induces key transcripts of pathways, which are associated with alternative activation as well as activation of the LXR pathway (**Figures 5B–E**). Interestingly, we reveal that while induction of *PPARγ* and *ORM-1* by sCD83 is dependent on LXR signaling activity (**Figure 5B**, first and second bar graphs), we observed that sCD83 upregulation of *KLF-4* is independent of LXR pathway inhibition (**Figure 5B**, third bar graph). This finding is in line with literature suggesting that KLF-4 is upstream of LXR (27). Of course, we also examined the expression of various surface molecules, such as MHC-II, MSR-1, CD14, CD163, and CD83 (**Supplementary Figure 2**). In this case, we were able to show that sCD83 regulates the expression of MSR-1, CD163, and CD83 (**Supplementary Figure 2**) independently of the LXR pathway, as it does for KLF-4 (**Figure 5B**). Next, we verified the observed transcriptional changes for ORM-1 and PPAR γ  also on the protein level using Western blot analyses. As depicted in **Figure 5C**, both ORM-1 and PPARγ are induced by sCD83 and this effect is reversed by blocking the LXR pathway. We also tracked the expression of target genes of PPARγ, such as *CD36*, *FABP4*, and *LXRa* as in our previous experiments; we observed an upregulation of all target genes, which were dependent on LXR signaling activity (**Figures 5D, E**). Since we observed that key transcription factors PPARγ and LXR involved in lipid metabolism are regulated by LXR signaling activity, we next checked Mφ for lipid droplet loading using BODIPY staining, which is a fluorescent dye that specifically stains lipid droplets in cells. As depicted in **Figure 5F**, we detected significantly less lipid load in Mφ that were treated with sCD83 indicating a rather pro-resolving metabolic state. However, this effect was independent of LXR activation (**Figure 5F**). On a final note, our data indicate that sCD83 induces a rather anti-inflammatory Mφ phenotype, function, and metabolic state, which is partially dependent on activation of the LXR pathway, but some effects are independent of LXR and other pathways might be involved.

## 3 Discussion

Adaptations of Mφ to diverse environmental cues are crucial for organ homeostasis, efficient clearance of pathogenic infections, and resolution after inflammation (28). However, an imbalance in Mφ biology, such as an excessive Mφ activation, can lead to impaired tissue homeostasis and concomitant development of chronic inflammatory diseases, such as multiple sclerosis or rheumatoid arthritis. Therefore, it is of utmost importance to develop new therapeutics for inflammatory diseases that modulate Mφ in a proper inflammation-resolving profile.

**Figure 6:**
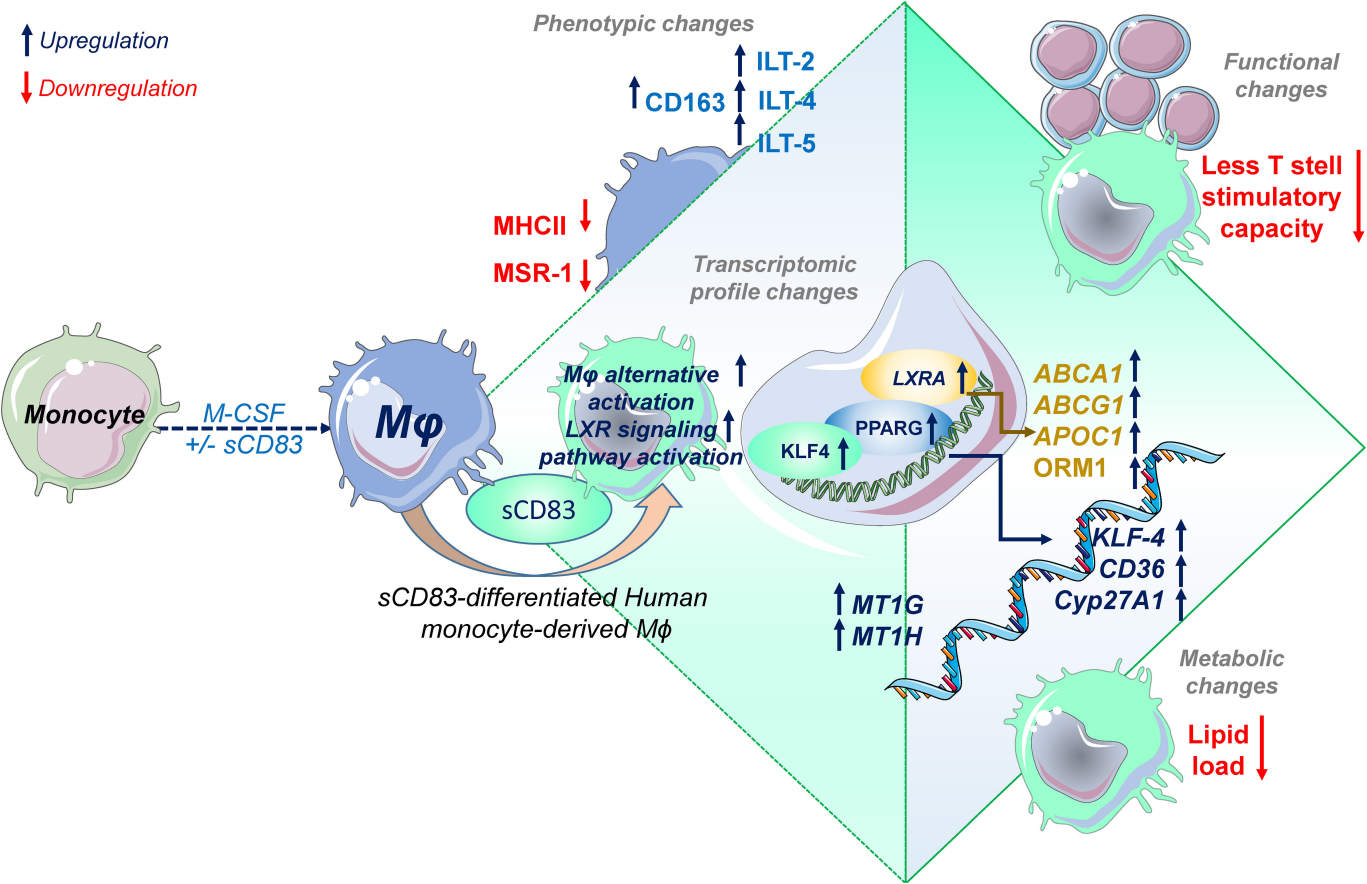
Soluble CD83 induces phenotypical, functional, and metabolic changes in human monocyte-derived Mφ. Administration of sCD83 to Mφ differentiation results in upregulation of pro-resolving receptors (CD163, ILT-2, ILT-4, ILT-5), whereas activation markers including MSR-1 or MHC-II are downregulated, which results in less T-cell stimulatory capacity of sCD83-differentiated Mφ. Administration of sCD83 to Mφ differentiation results in profound changes in transcriptome, including induction of pathways associated with alternative activation as well as liver X receptor pathway activation in Mφ. To this end, Mφ treated with sCD83 show increased expression of transcription factors KLF4, PPARG, and LXRA, which are characteristic for human alternatively activated Mφ. In line with that, sCD83-treated Mφ show enhanced levels of well-characterized target genes, such as *CD36*, *KLF-4*, *CYP27A1*, *ABCA1*, *ABCG1*, and *ORM1*, indicating modulation of the lipid metabolic state of sCD83-differentiated Mφ, which results in less lipid load. In summary, we present for the first time data on modulation of human Mφ by sCD83, which represents a further preclinical step for the development of sCD83 as a new therapeutic agent for the treatment of inflammatory conditions.

One such candidate, which might be beneficial to treat inflammatory conditions via the modulation of Mφ biology, is the soluble CD83 protein, which is the extracellular domain of the membrane-bound CD83 protein (mCD83). The immunomodulatory properties of sCD83 have been proven in several preclinical studies in the context of autoimmune disorders and transplantation procedures (11, 12, 23, 29–35). Recently, we proved that sCD83 modulates murine Mφ biology toward a regulatory phenotype, resulting in improved induction of tissue tolerance after corneal transplantation (12). In addition, sCD83 accelerated skin wound healing after systemic as well as topical treatment via the induction of pro-resolving Mφ (13). Notably, in a recently published study, Gong et al. suggest that porcine respiratory syndrome virus (PRRSV) induces sCD83 secretion by DCs thereby modulating polarization toward AAMs (14). In this study, we extended the investigation of sCD83-mediated effects on human Mφ and disclosed a striking modulation of Mφ phenotype, function, and metabolism. We observed that sCD83 administration to Mφ differentiation results in downregulation of MHC-II (**Figure 2A**) and MSR-1 (**Figure 2B**), suggesting the development of a pro-resolving Mφ phenotype (36). Interestingly, MHC-II expression has been reported to remain unaffected by sCD83-treated murine Mφ, highlighting the importance of the translational approach of the present study (11, 12). The expression of MSR-1 on human Mφ is associated with pro-inflammatory features, since triggering MSR-1 with specific ligands leads to AAM-CAM phenotypic switch and MSR-1 participates in the pathogenesis of multiple inflammatory diseases (37–39). Thus, the observed downmodulation in sCD83-treated Mφ suggests a less pro-inflammatory activity of these cells. Contrarily to MHC-II and MSR1, we observed a significant upregulation of the scavenger receptor CD163, which is a marker for regulatory anti-inflammatory Mφ induced upon glucocorticoid, IL-6, or IL-10 treatment (16). CD163 is also important for uptake of hemoglobin–haptoglobin complexes, which arise after tissue injury due to hemolysis of erythrocytes and subsequent binding of haptoglobin to released hemoglobin. The scavenging of hemoglobin–haptoglobin by CD163 is absolutely crucial for resolution of inflammation (40). We also observed a significant upregulation of ORM-1 in sCD83-differentiated Mφ (**Figure 4**). ORM-1 is known to induce polarization of monocytes toward an alternative activation state and upregulation of CD163 (18), which might account for the increased levels of CD163 (**Figure 2B**). Additionally, sCD83 administration to Mφ differentiation strikingly upregulates members of the leukocyte immunoglobulin-like receptor subfamily B (LILRB), namely, ILT-2, ILT-4, and ILT-5 (**Figures 2E–G**), which counteract inflammatory responses and are typically expressed on regulatory AAM (41–43) or monocytes stimulated with anti-inflammatory cytokine IL-10 (44, 45). Since we observed profound regulatory phenotypic changes in human Mφ when sCD83 was present during the differentiation process, we next assessed the functional consequences using an MLR assay. We observed a significantly reduced capacity of Mφ to stimulate alloreactive T cells when sCD83 was present during cell differentiation (**Figure 3**), further substantiating our data that sCD83 modulates human Mφ toward a less inflammatory phenotype. This is also in line with data from our previous work using murine Mφ showing that sCD83 administration reduced the capacity to stimulate allogeneic T cells *in vitro* (12). Subsequent bulk RNA sequencing analyses unraveled the sCD83-induced mechanisms and signaling pathways in human Mφ biology that reprogram Mφ toward regulatory phenotype and function. Pathways that were associated with inflammatory Mφ polarization, such as cytokine storm-induced signaling pathway and HIF1-α as well as IL-6 signaling, were significantly downregulated in sCD83-treated Mφ (**Figure 4B**). Accordingly, we observed significant reduction of HIF-1α target gene *EGLN3* (**Figure 4C**). At the same time, Mφ alternative activation signaling pathway and LXR activation were significantly induced in Mφ treated with sCD83. Therefore, we tracked the regulation of specific genes, which are involved in alternative activation as well as LXR activation. Strikingly, we observed a significant induction of key transcription factor KLF-4 on mRNA and protein levels by sCD83 in human Mφ (**Figure 4**). This transcription factor not only blocks polarization of CAM, e.g., by inhibiting NFĸB, but is also known to be crucially involved in reprogramming toward AAM (17, 46). Next to KLF-4, PPARγ is another master regulator for polarization of Mφ toward an anti-inflammatory profile (22, 47), which was also strikingly induced upon sCD83 administration to human Mφ differentiation (**Figure 4**). Given the fact that KLF4 deletion results in low PPARγ levels (17), it suggests that KLF-4 augments PPARγ expression indicating they cooperatively interact to stabilize AAM phenotype, function, and metabolism (17, 48). PPARγ is also a master transcription factor for fatty acid metabolism and directly regulates the expression of genes, such as *CD36*, *Cyp27A1*, *FABP4*, and *LXRa*, involved in lipid uptake transport and metabolism (49, 50). CD36 is important for fatty acid and cholesterol influx via receptor-mediated endocytosis and the increased lipid load is counterbalanced by increasing the expression of cholesterol efflux transporters, such as ABCG1 and ABCA1 via transcription factor activity LXRa. Expectedly, our data revealed that administration of sCD83 to Mφ differentiation results in significantly increased levels of the transcription factor LXRa, which is regulated by PPARγ, and its target genes *ABCG1*, *ABCA1*, and *APOC1* (**Figure 4I**). As shown by our LXR blocking experiments, the effects on activity of PPARγ depend on LXR signaling activity since blockade of this pathway reversed the sCD83-induced upregulation of PPARγ and the respective target genes (**Figures 5B, F**). Consequently, both transcription factors are known to control transcriptional programs involved in lipid uptake, efflux, lipogenesis, and metabolism and also negatively regulate programs that are associated with inflammatory responses (51). Indeed, we observed a decreased lipid load in Mφ that were treated with sCD83, indicating an altered lipid metabolic state, but this effect was not reversed by blocking the LXR pathway (**Figure 5I**). However, given the fact that pro-inflammatory CAM are characterized by increased lipid load (**Figure 5F**), we suggest that sCD83 also affects pathways other than LXR signaling, which reprogram Mφ toward anti-inflammatory phenotype, function, and metabolism (52). The finding that sCD83-induced expression of KLF4 and modulation of MSR-1, CD163 as well as CD83, is not reversed upon LXR-inhibition further corroborates this notion. In addition, our MLR assays also showed that sCD83-induced effects were not dependent on LXR activation (data not shown). Consequently, future studies are necessary to unravel the detailed metabolic changes and pathways induced by sCD83 in Mφ. In conclusion, within this study, we present for the first time data regarding phenotypic, functional, and metabolic changes in human Mφ biology by sCD83 (summarized in **Figure 6**).

## 4 Materials and methods

### 4.1 Generation of human monocyte-derived Mφ and treatment with sCD83

Human monocyte-derived Mφ were differentiated from peripheral blood mononuclear cells (PBMCs) isolated from Leukocyte Reduction System Chambers (LRSCs) from healthy blood donors via density gradient centrifugation using Lymphoprep (Nycomed Pharma). After isolation of PBMCs, 80 × 10^6^ PBMCs were seeded in 10-cm² dishes (Cat. No. 664-160, Greiner Bio-One) to allow monocyte adherence in adherence medium [RPMI 1640 (Lonza), 1% human AB serum (Sigma Aldrich), penicillin–streptomycin–glutamine solution (Sigma-Aldrich), and 10 mM HEPES (Lonza)], for 75 min. After adherence of monocytes, the non-adherent fraction (NAF) was collected and frozen for mixed lymphocyte reaction (MLR) assays (described below). To discard all non-adherent cells, dishes were washed three times with 10 ml of pre-warmed RPMI 1640 without supplements. Subsequently, monocytes were differentiated into human Mφ in differentiation medium [RPMI 1640 (Lonza), 10% human AB serum (Sigma-Aldrich), penicillin–streptomycin–glutamine solution (Sigma-Aldrich), 10 mM HEPES (Lonza)] and recombinant M-CSF (20 ng/ml, PeproTech). Fresh medium and recombinant M-CSF (20 ng/ml, PeproTech) were added on day 3. To analyze the effect of sCD83 on human monocyte-derived differentiation, sCD83 (25 µg/ml) or the corresponding amount of PBS was added as a control on day 0 as well as on day 3. On day 6, human monocyte-derived Mφ were harvested by using a cell scraper (Sarstedt, Cat. No.: 83.1830) and used for subsequent phenotypical and functional analyses.

### 4.2 Polarization of human monocyte-derived Mφ

After harvesting Mφ on day 6, Mφ were seeded in uncoated 24-well plates at a cell density of 2 × 10^6^ per ml and subsequently stimulated using LPS (100 ng/ml, Sigma-Aldrich) + IFN-γ (300 U/ml), or IL-4 (20 ng/ml) or left untreated for 16 h and used for subsequent phenotypical (flow cytometry) and functional analyses (MLR assays).

### 4.3 Inhibition of LXR pathway

To check whether sCD83-induced effects on murine monocyte-derived Mφ are dependent on LXR pathway activation, we blocked the LXR pathway by using an LXR antagonist named GSK2033 (Cat. No. HY-108688, MedChemExpress). Human monocyte-derived Mφ were generated from PBMCs, and the GSK2033 (1 mM) was applied on day 0 and day 3 to the differentiation process 1 h before sCD83 (25 µg/ml) was administered. On day 6, cells were harvested and flow cytometric analysis was performed to assess expression of surface receptors and lipid load, qPCR for expression of specific target genes, and Western Blot analyses (see below).

### 4.4 Flow cytometry

Extracellular staining of surface molecules on human Mφ was performed at 20 min at 4°C in PBS using the following antibodies: CD86 (Y1/82A) CD83 (HB15e), CD14 (63D3), MSR-1 (7C9C20), MHC-II (L243), CD11b (M1/70), CD163 (GHI/61), ILT-4 (27D6), ILT-5 (MKT5.1), ILT-3 (ZM4.1, eBioscience), ILT-2 (HP-F1, eBioscience), and CD36 (5-271). All antibodies were purchased from BioLegend and if not otherwise stated. For live–dead discrimination, we used either 7-AAD or Live/Dead Aqua. After staining, cells were washed in DPBS (1,500 rpm, 4°C, 3 min). After removing the supernatant, the cell pellet was diluted in PBS and cells were analyzed using the FACSCanto II (BD). The BODIPY staining procedure was performed before surface antibody staining. BODIPY was diluted 1:4,000 in DPBS, and Mφ were incubated for 15 min at room temperature in the dark. Afterward, cells were washed in DPBS (1,500 rpm, 4°C, 3 min). After removing the supernatant, extracellular staining of surface molecules was performed as described above. The signal of BODIPY was quantified via flow cytometry. Data were evaluated using the FlowJo software.

### 4.5 RNA extraction, cDNA synthesis, and qPCR

Total RNA was isolated from human monocyte-derived Mφ using the RNeasy Plus Mini Kit (Qiagen) according to the manufacturer’s instructions. Subsequently, 500 ng of total RNA was reversely transcribed using the First-Strand cDNA Synthesis Kit (#K1612, Thermo Fisher Scientific), as described by the manufacturer. To analyze mRNA expression levels for phenotypic analyses of huMφ and verification of RNA sequencing data, qPCR analyses were performed using the SYBR Green Super Mix (Biozym) on a CFX96 Real-Time system (Bio-Rad) and normalized to the reference gene transcript *RPL13A*. For primer sequences, see **Table 1**.

**Table 1:** Human primer sequences used in qPCR experiments (Sigma Aldrich).

Gene	Orientation	Sequences
*ABCA1*	Forward Reverse	5’-GGGCCTCGTGAAGTATGGAG-3‘ 5’-GCCATCCTAGTGCAAAGAGC-3‘
*ABCG1*	Forward Reverse	5’-ATGGCCGCTTTCTCGGTC-3‘ 5’-GTTGCTGGACACCACCTCAT-3‘
*APOC1*	Forward Reverse	5’-CAGGAAGATTGAGAGAGTGCCCC-3‘ 5’-TCCTTCAGCTTATCCAAGGCAC-3‘
*CD36*	Forward Reverse	5’-AGGACTTTCCTGCAGAATACCA -3‘ 5’-ACAAGCTCTGGTTCTTATTCACA -3’
*EGLN3*	Forward Reverse	5’-AGAGGTCTAAGGCAATGGTG-3’ 5’-TCTGGAAATATCCGCAGGATC-3’
*FABP4*	Forward Reverse	5’- ACTTGTCTCCAGTGAAAACTTTG -3’ 5’- GATCACATCCCCATTCACAC -3’
*LXRA*	Forward Reverse	5’- TCTGGACAGGAAACTGCACC -3‘ 5’-CCGCAGAGTCAGGAGGAATG -3‘
*KLF4*	Forward Reverse	5’-TGCGGCAAAACCTACACAAAG-3‘ 5’-GTTCATCTGAGCGGGCGAAT-3‘
*MT1G*	Forward Reverse	5’- CTAGTCTCGCCTCGGGTTG-3’ 5’- GCAGCTGCACTTCTCCGAT-3’
*MT1H*	Forward Reverse	5’-TTCTCGCTTGGGAACTCCAG-3‘ 5’-AGGAGCCACCAGCCTCG-3‘
*PPARG*	Forward Reverse	5’-GCCGTGGCCGCAGATTT-3‘ 5’-GGGAGTGGTCTTCCATTACGG-3‘
*GLUT1*	Forward Reverse	5’-CTGCTCATCAACCGCAAC-3‘ 5’-CTTCTTCTCCCGCATCATCT-3‘
*GLUT3*	Forward Reverse	5’–CAGCGAGACCCAGAGATG-3‘ 5’-TTGGAAAGAGCCGATTGTAG-3‘
*ORM-1*	Forward Reverse	5’–TTGCTTTTGACGTGAACGATGAG-3‘ 5’-TGCTTCTCCAGTGGCTCACA -3‘
*TREM-1*	Forward Reverse	5’-GTGGATGCTCTTTGTCTCAG-3’ 5’-GCATCTCTCCGTCCCTTATTATC-3’

### 4.6 MLR assays

Human monocyte-derived Mφ were generated in the presence of absence of sCD83 from different healthy donors. Subsequently, Mφ were seeded in 96-well plates in technical triplicates for stimulation via LPS+IFN-γ or IL-4 or left unstimulated for 16 h. Then, Mϕ were cocultured at different ratios with allogeneic NAF cells (400,000 cells per well) for 72 h (37°C, 5.5% CO_2_). To analyze the allogeneic T-cell proliferation capacity, cell cultures were subsequently pulsed with [^3^H]-thymidine (1 μC/well; PerkinElmer, Germany) for an additional 8–16 h. Culture supernatants were harvested onto Glass Fiber Filter Mates using an ICH-110 harvester (Inotech, Switzerland), and filters were counted in a 1450 microplate (Wallac, Finland). Cells of cocultures were also harvested after 72 h and used for flow cytometric analyses to determine frequencies of different T-cell subsets.

### 4.7 RNA sequencing and bioinformatics

RNA was isolated from human monocyte-derived Mφ that were differentiated +/− sCD83 using the RNeasy Plus Mini Kit (Qiagen) according to the manufacturer’s instructions. To remove residual DNA, an additional DNAse digestion step was included. Briefly, after RNA binding to RNeasy Spin columns, DNAse was added to the column and incubated (15 min, RT) to discard residual DNA. Then, RNA was purified according to the protocol. Afterward, bulk mRNA sequencing was performed by Novogene and analyzed as described: Raw paired end RNA sequencing reads were mapped to the reference genome obtained from Ensembl (GRCh38 103) using Rsubread (v. 2.6.4) within the R programming language with standard parameters. Gene-level counts were quantified using featureCounts from Rsubread. The DESeq2 package was used to calculate differentially expressed genes between the treatment and control groups using a two-factor design. Transcriptomic data were analyzed using the Ingenuity Pathway Analysis (IPA) software from QIAGEN.

### 4.8 Western blot

To assess protein levels of KLF-4, PPAR-γ and ORM-1 in whole-cell lysates of human monocyte-derived Mφ-differentiated +/−sCD83, Western blot analyses were performed. Whole-protein lysates (30 µg per lane) were separated via SDS polyacrylamide gel electrophoresis and blotted onto a nitrocellulose membrane (GE Healthcare). After blocking in blocking reagent (5% BSA-TBST), membranes were incubated with the following primary antibodies overnight (4°C): mouse-anti-human KLF4 (Cat. No.: sc-166100; Clone: B-9, Santa Cruz), mouse-anti-human PPAR-γ (Cat. No.: MA5-15417; Clone: 3A4A9, 1E6A1; Invitrogen), and mouse-anti-human ORM-1 (Cat. No. MA5-41544; Clone: D1; mouse β-actin (Clone: AC-74, Sigma-Aldrich). Specific signals were detected using the appropriate HRP-labeled secondary antibody and the ECL Prime Western Blotting Detection Reagent (GE Healthcare). Quantification of Western blots was performed using the ImageJ/Fiji software (53). The intensities of bands are visualized in bar graphs and represent the protein amount in arbitrary units. Band intensities of KLF-4, PPARγ, and ORM-1 were normalized to β-actin that served as a loading control.

### 4.9 Statistical analyses

All statistical analyses were performed using GraphPad Prism 9.3.1. Statistical analyses were performed by two-way ANOVA, one-way ANOVA, or the appropriate corresponding non-parametric test. As stated, wherever necessary, we used non-parametric tests (Mann–Whitney U) when data were not normally distributed. Data are presented as mean values including the standard error of the mean (SEM). p-values of *p<.05; **p<.01; ***p<.001; and ****p<.0001 were considered statistically significant.

## Data availability statement

The data presented in the study are deposited in the Gene Expression Omnibus (GEO) repository, accession number GSE245761.

## Ethics statement

The studies involving humans were approved by Ethik-Komission der Friedrich-Alexander Universität Erlangen-Nürnberg (431_19B). The studies were conducted in accordance with the local legislation and institutional requirements. The participants provided their written informed consent to participate in this study.

## Author contributions

KP-M: Writing – original draft, Writing – review & editing, Visualization. Supervision. AW: Writing – review & editing, Investigation. LSp: Writing – review & editing, Investigation. MF: Writing – review & editing, Investigation, Data curation. PB: Writing – review & editing, Investigation. JPA: Writing – review & editing, Investigation. PS: Writing – review & editing, Investigation. AStr: Writing – review & editing, Investigation. PM-Z: Writing – review & editing, Investigation. NR: Writing – review & editing, Investigation. MK: Writing – review & editing. GK: Writing – review & editing. LSt: Writing – review & editing, Investigation. ASte: Writing – review & editing, Supervision. DR: Writing – review & editing, Supervision.
