# Supplementary material for: Soluble CD83 modulates human-monocyte-derived macrophages toward alternative phenotype, function, and metabolism
Source: Front Immunol. 2023 Dec 14;14:1293828. doi: 10.3389/fimmu.2023.1293828 (PMC10755915; doi:10.3389/fimmu.2023.1293828)
Supplement: Supplementary file 1 [file DataSheet_1.docx]

Supplementary Material

Soluble CD83 modulates human-monocyte-derived macrophages towards alternative phenotype, function and metabolism

Katrin Peckert-Maier^1^, Andreas B. Wild^1^, Laura Sprißler^1^, Maximilian Fuchs^3^, Philipp Beck^1^, Jean-Philippe Auger^2^, Pia Sinner^1^, Astrid Stack^1^, Petra Mühl-Zürbes^1^, Ntilek Ramadan^1^, Meik Kunz^3^, Gerhard Krönke^2^, Lena-Stich^1^, Alexander Steinkasserer^1^, Dmytro Royzman^1^

^1^Department of Immune Modulation, Universitätsklinikum Erlangen, Friedrich– Alexander Universität Erlangen–Nürnberg, 91052 Erlangen, Germany

^2^Department of Internal Medicine 3 – Rheumatology and Immunology, Friedrich-Alexander University Erlangen-Nürnberg (FAU) and Universitätsklinikum Erlangen, Erlangen 91054, Germany

^3^Fraunhofer Institute for Toxicology and Experimental Medicine (ITEM), Hannover, Germany

*** Correspondence:** Katrin.peckert@uk-erlangen.de

## Supplementary Figures


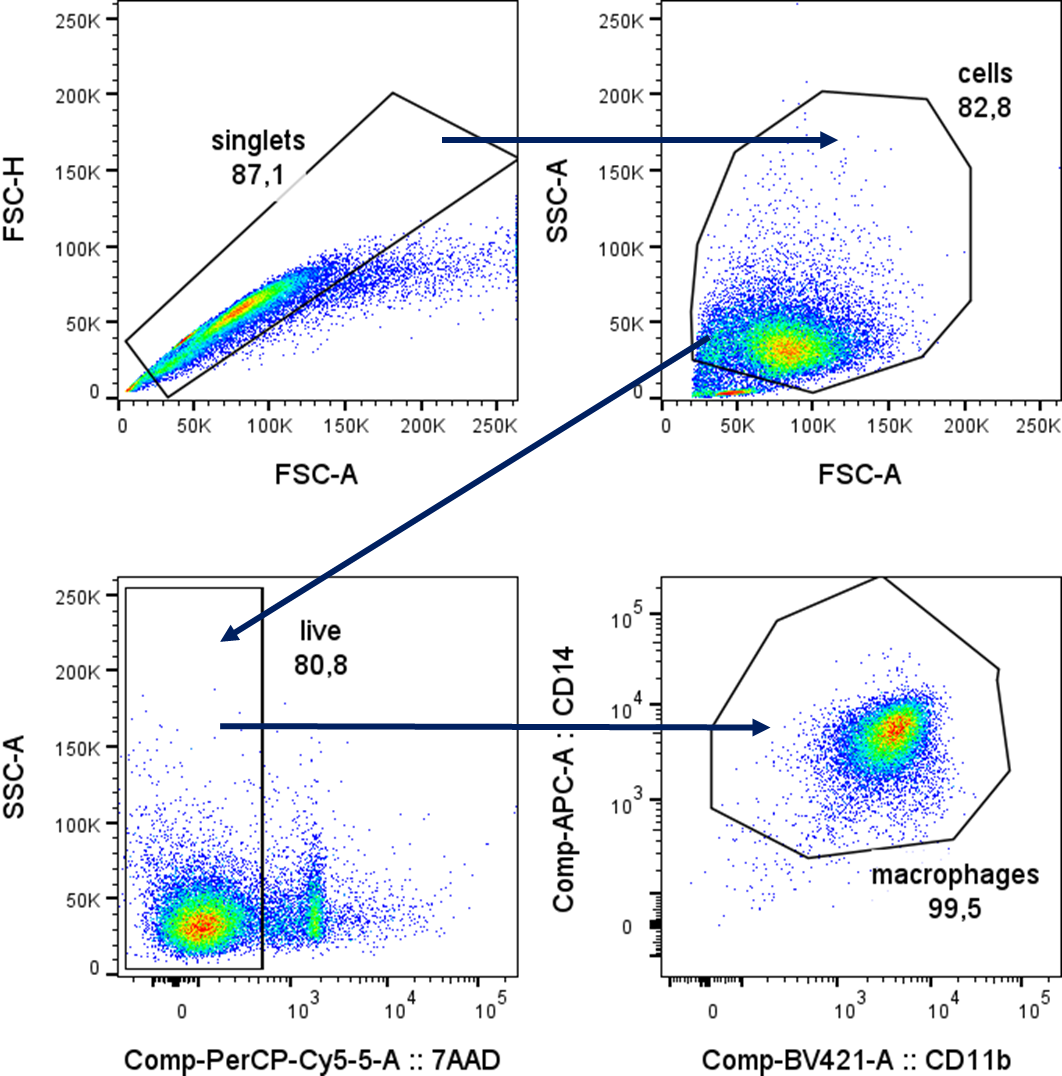


**Supplementary Figure 1:** **Gating strategy for the analyses of human monocyte-derived Mϕ** At first douplet cells were excluded to gate on single cells. Subsequently cell population was gated using SSC and FSC. Subsequently, dead cells were excluded by gating on the 7-AAD- negative population. Lastly Mφ were identified using a CD14+ CD11b+ gate. 20,000 events were measured within the CD14 – CD11b gate to analyze expression of different molecules on human macrophages.

**Supplementary Figure 2:** **Soluble CD83 induced modulation of surface receptors are independent of LXR pathway activation.** PBMCs were isolated via density gradient centrifugation and subsequently monocytes were seeded for adherence. Monocytes were differentiated into Mφ in the presence of M-CSF (20 ng/ml), +/- sCD83 (25 µg/ml). sCD83 was added on day 0 as well as day 3 during the differentiation process. GSK2033 (1mM) was applied one hour before sCD83 administration. Mφ were harvested on day 6 and subsequently analyzed by flow cytometry for the expression of CD14, MHC-II, CD163, MSR-1 and CD83. Gating strategy is displayed in S1**.** Data are represented as mean ± SEM. Statistical analysis was performed using a One-way ANOVA. Experiments were performed at least three times. One dot per bar graph represents one donor. n.s., not significant, which indicates there is no statistical significance; * p<0.05; ** < 0.01; *** p < 0.001; **** p < 0.0001.
